# Bio-Inspired Thermoregulatory Textile Enabled by Flexible Bidirectional Shape Memory Polymer

**DOI:** 10.3390/biomimetics11050345

**Published:** 2026-05-15

**Authors:** Jing Yan, Yahong Wang, Zhaoyang Wang, Yiqi Zhang, Yitian Zhou, Vitali Lipik, Guang Yang

**Affiliations:** 1 School of Textile Science and Engineering, Tiangong University, Tianjin 300387, China; yanjing@tiangong.edu.cn (J.Y.);; 2 Jiangsu Textile Products Quality Supervision and Inspection Institute, Nanjing 210007, China; 3 China Knitting Industrial Association, Beijing 100020, China; 4 School of Material Science and Engineering, Nanyang Technological University, 50 Nanyang Avenue, Singapore 639798, Singapore

**Keywords:** thermoregulatory textile, environmental adaptability, shape memory polymer, wearing comfort

## Abstract

Passive thermoregulatory textiles, operating without external energy input, play a crucial role in maintaining the human body within the thermal comfort zone. However, integrating autonomous environmental adaptability with superior wearing comfort into a single textile remains a challenge. In this work, inspired by the autonomous actuation of water lilies, we proposed an intelligent strategy to fabricate thermoregulatory textiles that dynamically adapted to ambient temperature fluctuations, driven by a bidirectional shape memory polymer (SMP). To concurrently achieve robust thermal adaptability and human-body-compatible softness, a crosslinked polyethylene glycol–butyl acrylate (PEG-BA) bidirectional SMP network was engineered. The PEG phase, featuring a broad crystal size distribution, provided the dynamic skeleton for thermally induced actuation, while the incorporation of the BA component tuned the intrinsic softness to match conventional soft textiles. Consequently, the synthesized PEG-BA network exhibited an exceptional bidirectional shape memory effect with a reversible strain of 15.5%, while maintaining high macroscopic softness comparable to that of human skin. By integrating this bidirectional polymer into a garment to form adaptive vents, the smart textile demonstrated the capability to significantly elevate human thermal comfort. Specifically, the vents autonomously open in hot environments to accelerate heat dissipation and close in cool environments to suppress heat loss. Given its exceptional personal thermoregulatory performance and wearing compliance, this proposed strategy exhibits considerable potential for maintaining optimal human comfort against fluctuating environmental conditions.

## 1. Introduction

Thermal comfort is a fundamentally important factor for both human physiological well-being and psychological health. As the primary boundary layer between the human body and external environment, garments play a critical role in managing heat transfer to sustain a comfortable microclimate [1,2,3,4]. In daily life, individuals frequently encounter sudden environmental temperature fluctuations, such as walking from air-conditioned indoor spaces to hot outdoor settings. Conventional fabrics lack the capacity to dynamically adapt to these rapid thermal changes, often resulting in thermal discomfort [5]. Therefore, there is an urgent need to develop intelligent, environmentally adaptive textiles that can autonomously ensure continuous wearer comfort across varying climates [6,7,8].

Current thermoregulatory textiles are generally categorized into active and passive types. Although active types can offer active control on thermal management, their dependence on external power sources (e.g., electricity) restricts their practicality for daily wear [9,10]. Passive types, which operate without external energy, are generally more desirable, especially for bidirectionally (i.e., heating and cooling) responsive textiles [11,12]. Phase change material (PCM)-based textiles are a typical example of bidirectional thermal management, which operate by absorbing heat for melting and releasing heat upon crystallization, consequently maintaining the textile temperature under exposure to changes in the environment temperature [13,14,15]. Nevertheless, the thermoregulatory capacity of PCMs is inherently transient, ceasing entirely once the phase transition is complete [16]. Given that heat exchange between the human body and the ambient environment is predominantly mediated by the worn fabric, developing textiles capable of dynamic structural reconfiguration presents a highly effective and sustainable alternative.

**Figure 1:**
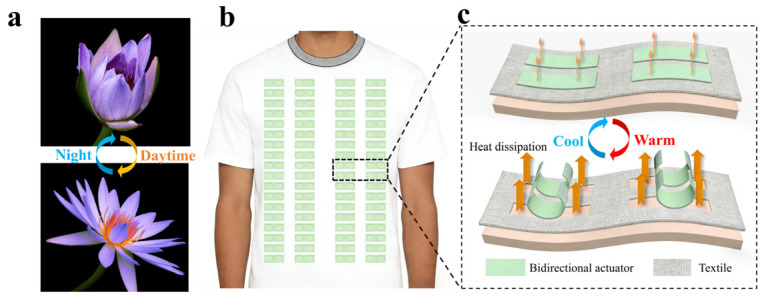
Conceptual illustration of thermoregulatory textile. (**a**) Dynamic nature of water lily petals, (**b**) structural design and (**c**) temperature-responsive actuation of thermoregulatory textile.

In nature, water lilies demonstrate a remarkable dynamic, stimulus-responsive functionality [17,18], gracefully opening their petals during daylight and closing them at nightfall, as vividly illustrated in Figure 1. Mechanistically, under abundant sunlight, the cells on the inner surface of the petals elongate more rapidly than those on the outer surface, driving the outward opening of the bloom. Conversely, the inner cells elongate slowly at night while the outer cells remain relatively stable, resulting in the inward closure of the petals [19]. Furthermore, this precise physiological actuation effectively minimizes thermal dissipation at night and protect itself against cold temperatures. Bidirectional shape memory polymers (SMPs) offer a synthetic analogue to this biological mechanism, capable of undergoing reversible shape changes upon exposure to specific environmental stimuli [20,21]. Based on molecular design strategies, thermally induced bidirectional SMPs can be generally classified into three categories: semi-crystalline polymer networks [22], liquid crystalline elastomer networks [23], and interpenetrating polymer networks [24]. Among them, semi-crystalline polymer networks have attracted significant attention due to their simple synthetic chemistry and the ease of tailoring their transition temperatures. The bidirectional shape memory effect in semi-crystalline networks stems from melting-induced contraction (MIC) and crystallization-induced elongation (CIE) [25]. Upon heating, aligned crystalline domains melt, driving macroscopic contraction via entropy recovery while the crosslinked network preserves structural integrity. Upon cooling, the network’s topological constraints and residual crystals acting as nucleation sites induce recrystallization along the original orientation. This directional crystallization forces polymer chains to extend, driving spontaneous macroscopic elongation. Therefore, leveraging SMPs as actuators to reversibly alter textile structures is thus a promising strategy for dynamic thermal management. Following this concept, Chai et al. [26] previously developed temperature-adaptive clothing utilizing a metallized shape memory copper/polyethylene film to modulate the opening and closing of the preset openwork. However, the intrinsic rigidity of such metallized shape memory composites severely mismatches the inherent softness of conventional fabrics, significantly compromising wearing comfort. Therefore, designing a temperature-responsive, mechanically tunable bidirectional SMP that perfectly matches the inherent softness of traditional textiles remains a critical challenge for maximizing both wearer comfort and thermoregulatory efficiency.

In this work, inspired by the autonomous actuation of water lilies, we proposed a smart, adaptive strategy to fabricate a passive thermoregulatory textile by integrating a highly flexible bidirectional SMP. To simultaneously achieve good thermal actuation and human-body-compatible softness, a bidirectional SMP network was engineered via the copolymerization of two key components: a crystalline polyethylene glycol (PEG) network functioning as the primary skeleton and a poly(butyl acrylate) (PBA) component. The strategic incorporation of the PBA segments precisely tuned both the thermal transition temperature (aligning it perfectly with the human physiological comfort zone) and the macroscopic softness (ensuring mechanical compatibility with conventional wearable textiles). Dynamic mechanical analysis (DMA) and macroscopic visual observations confirmed that the synthesized PEG-BA network exhibited an exceptional bidirectional shape memory effect (SME), delivering a remarkable reversible actuation strain of 15.5%. As a proof-of-concept, by integrating these PEG-BA polymeric actuators into a conventional cotton garment, we successfully developed a dynamic thermoregulatory prototype capable of autonomously regulating its engineered adaptive vents. Specifically, these rectangular flaps spontaneously opened in hot environments to accelerate heat dissipation and completely closed in cool environments to suppress convective heat loss, with exhibiting no actuation fatigue over multiple thermal cycles. This dynamic structural responsiveness highlights the tremendous potential of the bidirectional SMP network for next-generation, high-comfort, passive thermal management textiles.

## 2. Materials and Methods

### 2.1. Materials

Polyethylene glycol (PEG) with varied molecular weights (2000, 4000, and 10,000 g·mol^−1^), butyl acrylate (BA, ≥99%), 2-isocyanatoethyl methacrylate (2-IEM, ≥98%), and dibutyltin dilaurate (DBTDL, ≥95%) were purchased from Aladdin. 2,2-azoisobutyronitrile (AIBN), dichloromethane (DCM), and n-hexane were obtained from Macklin and dehydrated using molecular sieves prior to use. Conventional knitted cotton fabric was obtained from commercial sources.

### 2.2. Synthesis of PEG-BA Networks

The methacrylation of PEG was performed according to our previously reported procedure [27]. Taking PEG of 2000 g·mol^−1^ as an example, PEG (20 g, 10 mmol) was dissolved in 60 mL of DCM, followed by the addition of an excess of 2-IEM (3.2 g, 21 mmol) and DBTDL (0.1 g) as a catalyst. The reaction solution was magnetically stirred for 4 d at ambient temperature. Upon completion, the synthesized PEG dimethacrylate (PEGDM) was precipitated by adding excessive n-hexane. The resulting precipitate was filtered and subsequently dried under vacuum at 30 °C. The methacrylation of the other two PEG samples (4000 and 10,000 g·mol^−1^) was achieved by repeating these steps.

The crosslinked PEG-BA networks were fabricated via thermally triggered free-radical copolymerization [28]. To achieve a broad distribution of crystal sizes, the three PEGDM precursors (corresponding to the different molecular weights) were mixed in equal weight ratios at 70 °C, and then blended with varying contents of BA (10, 20, 40 wt%) and 0.3 wt% of AIBN as the radical initiator. The homogeneous precursor mixture was cast into a glass mold and polymerized for 24 h at 80 °C. To remove uncrosslinked elements, the resulting crosslinked films were purified in DCM and subsequently dried in a vacuum oven at 40 °C. The samples with different BA loadings were denoted as PEG-BA-x (where x is the mass percentage of BA relative to the total sample weight).

### 2.3. Fabrication of Thermoregulatory Textile

To construct the thermoregulatory garment, a commercial knitted cotton textile was engineered using laser-induced thermal cutting technology, creating rectangular flaps that remained hinged to the fabric at one edge, which simultaneously fused the cut edges to prevent yarn unraveling. Subsequently, rectangular strips of the temperature-actuated PEG-BA polymer film after programming were precisely integrated over these flaps using a flexible textile coating adhesive, yielding a thermoregulatory textile equipped with actuated flaps in response to temperature fluctuations.

### 2.4. Characterization

The methacrylation of PEG chains and the synthesis of the PEG-BA networks were characterized using Fourier-transform infrared (FTIR) spectroscopy (Nicolet iS20, Thermo Fisher Scientific). The FTIR spectra were recorded over a wavenumber range of 4000 to 600 cm^−1^, with 32 scans and a resolution of 4 cm^−1^. The crosslinking degree of the PEG-BA polymers was evaluated by the gel fraction and swelling ratio. Following purification in DCM, the gel fraction was defined as the mass ratio of the dried purified polymer to its original mass, while the swelling ratio was defined as the mass ratio of the maximally swollen gel to the dried purified polymer. Thermal transitions were investigated via differential scanning calorimetry (DSC, 204F1, Netzsch, Selb, Germany) under an N_2_ atmosphere. The polymer samples were subjected to a heating–cooling cycle between −40 °C and 100 °C at a ramp rate of 10 °C min^−1^. The degree of crystallinity (X) was calculated by dividing the endothermic peak area by 205 J g^−1^ of the theoretical melting enthalpy of a 100% crystalline PEG [29]. Mechanical properties were assessed using a universal testing machine (Model 3369, Instron, Norwood, MA, USA). The specimens with a width of 5 mm were tested, and the gauge length was 20 mm. Dynamic mechanical analysis (DMA, Q800, TA Instruments, New Castle, DE, USA) was performed in tension mode on rectangular samples of approximately 30 mm × 5 mm × 0.6 mm. The bidirectional shape memory effect (SME) was quantitatively evaluated via heating and cooling cycles. The polymer film was initially programmed through a two-step process: stretching the sample to a 20% strain at 70 °C and maintaining this deformation for 10 min, followed by rapidly quenching the sample to −20 °C for 10 min under constant stress to fix the temporary shape. Finally, after removal of the external stress, the programmed film was subjected to alternating thermal cycles between 37 °C and 0 °C to monitor the bidirectional SME.

After confirming the successful synthesis of the PEG-BA polymer, the thermoregulatory textiles were then fabricated. The macroscopic temperature-responsive behavior of the thermoregulatory textile was evaluated in an environmental test chamber (MQ-DTH50F-2N, ZhongKeMeiQi Technology Co., Ltd., Tianjin, China). Bending angle measurements of the actuated flaps were recorded at various ambient temperatures. Digital photographs were taken when the dynamic textile reached a steady equilibrium state at each targeted temperature.

## 3. Results and Discussion

### 3.1. Design Principle of Bio-Inspired Thermoregulatory Textile

Inspired by the dynamic stimulus-responsive nature of water lily petals, we designed an intelligent thermoregulatory textile by integrating programmed bidirectional SMPs as temperature-responsive actuators into a conventional textile to create adaptive vents, i.e., rectangular flaps (Figure 1b). Consequently, in a cool environment, these adaptive vents maintain their original flat, closed conformation to suppress body heat dissipation. Conversely, in a hot ambient environment, the flaps autonomously bend outward, thereby enhancing heat dissipation (Figure 1c). This dynamic and intelligent regulation of body heat significantly improves wearer comfort across fluctuating environmental conditions.

The operational mechanism of this smart thermoregulatory textile fundamentally relies on the designed bidirectional SMP-driven actuators. Specifically, these actuators have a heterogeneous bilayer architecture constructed by securely laminating the bidirectional SMP film onto the underlying textile substrate. Within this configuration, the outer bidirectional SMP serves as the active layer, capable of reversible elongation and contraction in response to temperature variations, whereas the textile layer acts as a temperature-inert constraint with negligible thermal expansion. Consequently, upon exposure to a hot environment, the outer SMP film undergoes macroscopic contraction while the inner textile layer maintains its original length. Driven by this thermally induced mechanical mismatch, the heterogeneous bilayer actuator bends outward toward the ambient environment. In this open state, body-heat dissipation is drastically amplified by facilitating the transmission of human infrared radiation to the surroundings, accompanied by synergistically enhanced air convection and sweat evaporation, thereby achieving a pronounced cooling effect. Correspondingly, in a cool environment, the thermally induced elongation of the SMP film forces the bilayer actuator to recover its original flat (closed) conformation. In this state, outward infrared radiation and convective heat exchange are effectively suppressed, thereby minimizing heat loss and keeping the human body warm. Therefore, to realize this dynamic thermoregulatory mechanism, the design of the bidirectional SMP is paramount, which requires not only a good bidirectional SME, but also an intrinsic softness that must perfectly match that of the highly compliant textile substrates to ensure the wearer’s tactile comfort.

### 3.2. Synthesis and Characterization of Bidirectional SMP Networks

**Figure 2:**
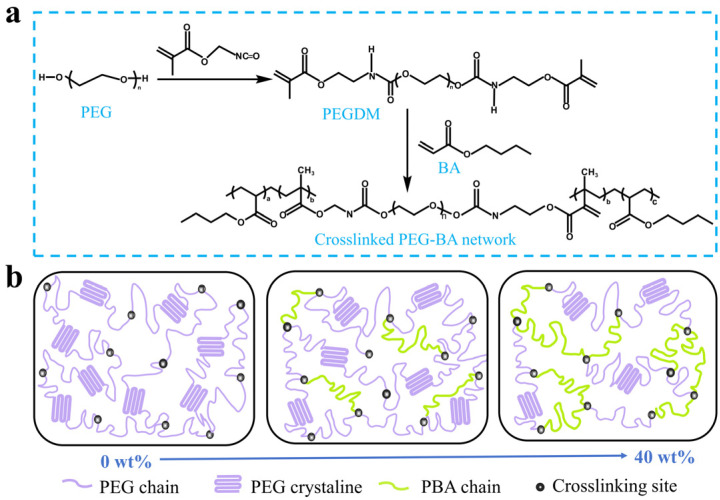
(**a**) Synthesis route and (**b**) structural design of crosslinked PEG-BA networks.

To realize the bidirectional SMP, a broad distribution in the crystal size, corresponding to a broad melting/crystallization temperature range, is a prerequisite. To achieve this broad crystalline distribution, crystalline PEG mixtures with varying molecular weights (i.e., 2000, 4000, and 10,000 g·mol^−1^) were employed as the primary polymeric skeleton according to our previous work [29]. Concurrently, the intrinsic flexibility of the polymer network was precisely modulated by incorporating varying contents of BA. The crosslinked networks of PEG-BA polymers were successfully constructed via the free-radical copolymerization of the acrylate groups in PEGDM and BA. The detailed synthetic route and the proposed structural models of the PEG-BA networks with different BA contents are illustrated in Figure 2.

**Figure 3:**
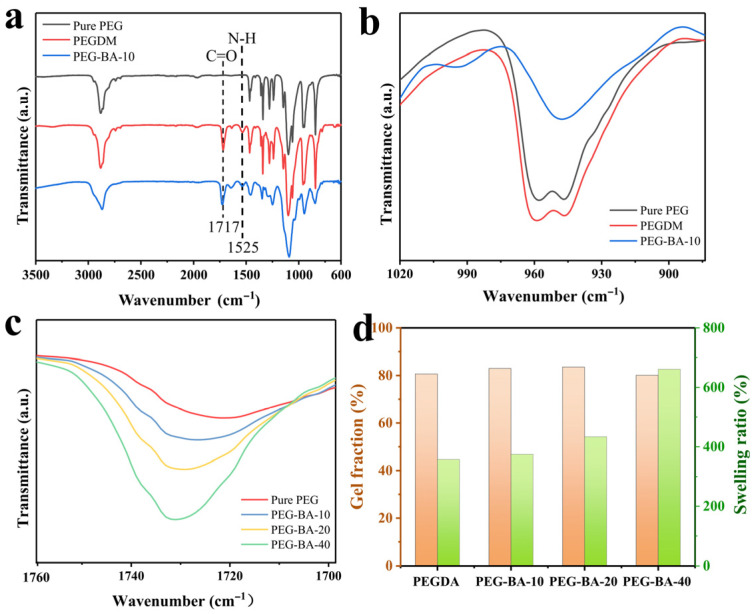
Characterizations of crosslinked PEG-BA networks. (**a**) FTIR spectra and (**b**) the magnified views of PEG, PEGDM, and PEG-BA-10, (**c**) FTIR spectrum comparison of PEG-BA networks with different BA contents, and (**d**) gel fraction and swelling ratio of different PEG-BA networks.

The successful synthesis of the PEG-BA networks was systematically confirmed via FTIR spectroscopy (Figure 3a). Compared to pristine PEG, the spectrum of PEGDM exhibited two new distinct peaks at 1717 cm^−1^ and 1525 cm^−1^. These peaks are attributed to the stretching vibrations of the C=O and N–H bonds, respectively, originating from the urethane linkages formed by the reaction between the terminal –OH groups of PEG and the –NCO groups of 2-IEM [30]. Furthermore, a pronounced enhancement of the broad peak centered at 950 cm^−1^ was observed for PEGDM, which is attributed to the overlapping vibrational bands of the methylene and acrylate groups [31]. Coupled with its subsequent attenuation after the crosslinking reaction, this change provides an evidence for the successful grafting of acrylate groups onto the PEG chains (Figure 3b). Subsequent thermally initiated free-radical polymerization yielded the crosslinked PEG-BA networks. The intensity of the acrylate peak at 950 cm^−1^ in the PEG-BA polymer significantly diminished compared to that of the PEGDM precursor, indicating the occurrence of addition polymerization. Additionally, as the BA content increased, it was observed that the intensity of the C=O stretching peak at 1730 cm^−1^ correspondingly intensified, verifying the progressive incorporation of the BA component into the polymer network (Figure 3c).

The structural integrity of the crosslinked PEG-BA networks was further evaluated through the gel fraction and swelling ratio, as presented in Figure 3d. The gel fractions for all synthesized samples, regardless of BA content, consistently exceeded 80%, demonstrating that highly efficient chemical crosslinking occurred and the majority of the precursors were successfully incorporated into the polymer network. The relative crosslinking density of the PEG-BA networks was qualitatively assessed using the swelling ratio. Notably, the swelling ratio of the PEG-BA networks exhibited an increase with higher BA loadings, from 357% for the pure PEG network to 661% for the PEG-BA-40 network. This upward trend indicated a progressive reduction in the overall crosslinking density as the BA content increased. This behavior occurred because PEGDM acted as the macroscopic crosslinker within the system, whereas the polymerization of BA yielded highly flexible linear poly(butyl acrylate) (PBA) chains. Consequently, the increasing amount of BA decreased the crosslinking density. This reduction in crosslinking density, synergistically coupled with the introduction of highly flexible linear PBA chains, plays a pivotal role in tuning the macroscopic softness of the polymer network, thereby ensuring its mechanical compatibility with highly compliant traditional textiles.

### 3.3. Crystallinity and Mechanical Performance of the PEG-BA Networks

**Figure 4:**
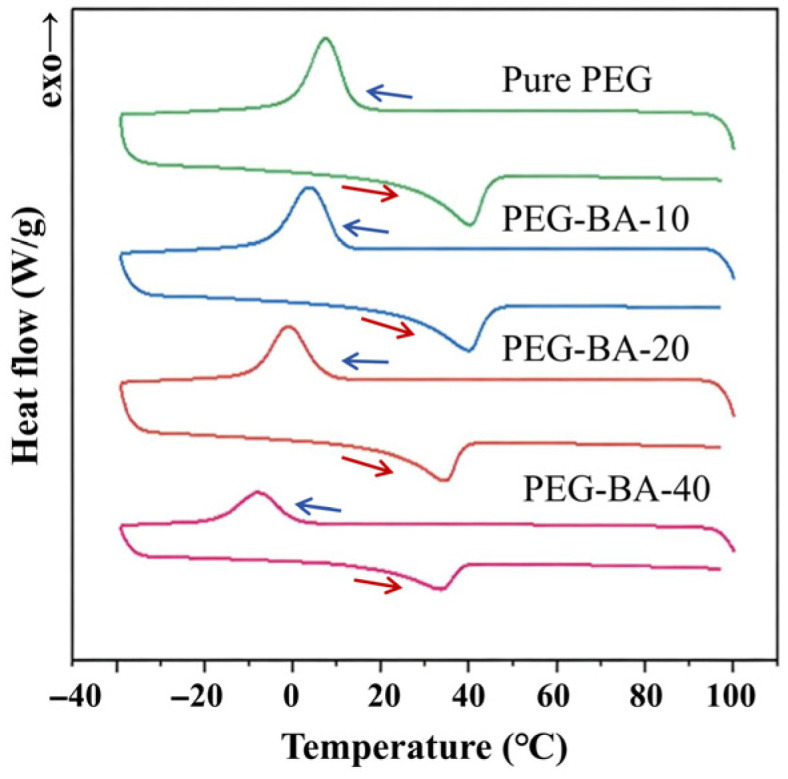
DSC curves of different PEG-BA networks under a heating–cooling cycle.

It is known that the bidirectional SME in crosslinked polymer networks fundamentally originates from the reversible melting and recrystallization of the internal crystalline phases [25]. Therefore, the DSC technique was employed to investigate the thermal transitions and crystallization behaviors of the PEG-BA networks, specifically to elucidate the influence of varying BA contents. Figure 4 presents the DSC curves under heating and cooling cycles for the synthesized PEG-BA polymers, and the corresponding thermal parameters, including the onset temperature (Tonset), peak maximum temperature (Tp), end temperature (Tend), melting enthalpy (ΔH), and degree of crystallinity (X), are summarized in Table 1.

Notably, designing an effective bidirectional shape memory actuator necessitates a broad crystal size distribution, which macroscopically manifests in a broad melting temperature range. To fulfill this physical requirement while simultaneously tuning the transition temperature to align with human body temperature, a strategic combination of PEG precursors with varied molecular weights was employed [28]. As expected, all PEG-BA samples exhibited a broad melting temperature range over 30 °C. Given that shape memory deformation is strictly driven by the melting of crystalline domains, such a broad melting range ensures that the polymer undergoes continuous and progressive actuation across this wide temperature window. Furthermore, the peak melting transitions are highly concentrated within the range of 34~42 °C, which are close to the physiological temperature of the human body and thus enabling localized personal thermal management. As the BA content increased from 0 to 40 wt%, the melting and crystallization peaks systematically shifted toward lower temperatures, while both the melting enthalpy and the calculated crystallinity decreased significantly. These thermal behaviors indicate the reductions in both the overall crystallinity and the average crystallite size within the polymer networks. This phenomenon is primarily attributed to the decreased weight fraction of the crystalline PEG segments, synergistically coupled with the steric hindrance imposed by the highly flexible linear PBA chains, which actively restricts the orderly packing and crystallization of the PEG segments. By leveraging this predictable variation in transition temperatures, the adaptive and intelligent actuation behavior of the PEG-BA polymers can be precisely tailored to meet the specific requirements of diverse wearable applications.

**Figure 5:**
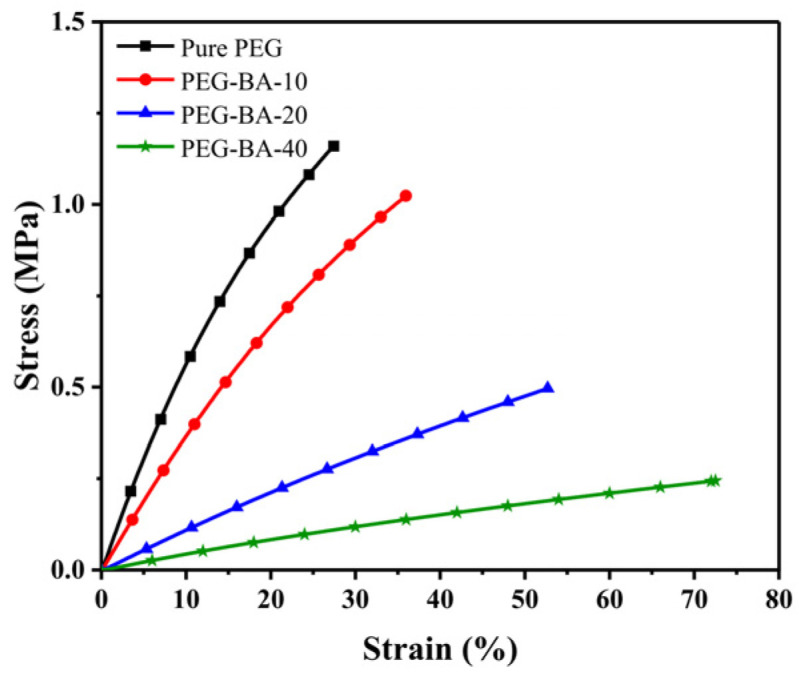
Typical stress–strain curves of the PEG-BA networks.

The mechanical properties of polymer networks are critical factors for wearable applications, particularly concerning wearer comfort. Furthermore, evaluating these properties reveals the maximum deformation, which provides essential guidance for the subsequent shape-memory programming process. The mechanical behaviors of all synthesized PEG-BA networks were systematically assessed via tensile testing, and the representative stress–strain curves are presented in Figure 5. Evidently, the pure PEG network exhibited a relatively rigid and brittle nature, characterized by a higher elastic modulus but a limited elongation at break of only 27.4%. However, the macroscopic softness of the polymer networks was progressively enhanced with the continuous incorporation of the BA component. Specifically, as the BA content increased from 0 to 40 wt%, the elastic modulus and the ultimate tensile strength dramatically decreased from 6.5 MPa to 0.45 MPa and from 1.2 MPa to 0.2 MPa, respectively. However, the elongation at break significantly increased from 27.4% to 71.7%. This pronounced softening phenomenon is fundamentally governed by three synergistic factors: the degree of crystallinity, the crosslinking density, and the intrinsic flexibility of the incorporated PBA chains. As previously elucidated, the progressive addition of BA inevitably weakened both the crystallization and the crosslinking density of the PEG domains. This microstructural disruption significantly enhanced the mobility of the polymer chains, directly contributing to the macroscopic reduction in tensile strength and the concurrent improvement in elongation at break. More importantly, given the inherently soft and elastomeric nature of linear PBA [32], a higher fraction of PBA domains fundamentally plasticized the bulk material, further softening the entire crosslinked network. Consequently, the synergistic interplay of reduced crystallinity, lowered crosslinking density, and the plasticizing effect of PBA segments endows the PEG-BA networks with highly tunable mechanical softness, perfectly satisfying the stringent comfort requirements for dynamic thermoregulatory textiles.

### 3.4. Bidirectional Shape Memory Behavior of the PEG-BA Networks

Considering the requirements for both a suitable transition temperature for the bidirectional SME and high macroscopic softness, the PEG-BA-20 network was selected as a representative model to systematically investigate its bidirectional shape memory behavior. This specific formulation was chosen because its thermal transition temperature falls perfectly within the human thermal comfort zone, and its elastic modulus closely matches that of human skin. The bidirectional SME of PEG-BA-20 was quantitatively evaluated via the DMA technique. The polymer films were initially programmed according to the following thermomechanical procedure: the specimens were heated to 70 °C to completely melt the crystalline domains, uniaxially stretched to 20% of their original length, and subsequently cooled to −10 °C under constant stress to fix the temporary shape. Interestingly, during this cooling process, a further spontaneous increase in strain was observed. This phenomenon is primarily attributed to the alignment of polymer chains and the subsequent oriented crystal growth along the stretching direction. Upon removal of the external stress, the shape fixity ratio of PEG-BA-20 was determined to be 84%. Following the programming phase, the bidirectional SME of the specimens was evaluated by subjecting them to stress-free heating and cooling cycles between 0 °C and 37 °C (the upper limit being specifically chosen to align with human body temperature).

**Figure 6:**
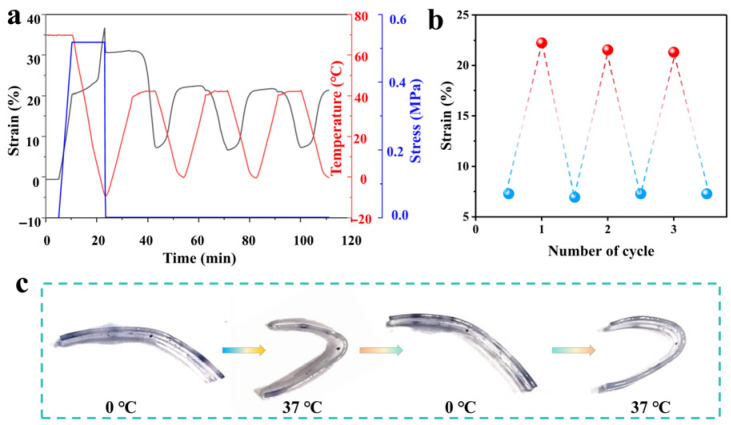
Bidirectional SME of the PEG-BA network. (**a**) DMA curves showing bidirectional shape memory behavior, and (**b**) its cyclic stability and (**c**) visual demonstration.

As illustrated in Figure 6a, the strain decreased significantly upon heating from 0 °C to 37 °C; conversely, the strain increased when the temperature was reversed from 37 °C back to 0 °C. This fully reversible, bidirectional actuation strain reached an impressive 15.5% and remained highly stable over multiple thermomechanical cycles (Figure 6b). The fundamental mechanism driving this thermally responsive bidirectional SME is governed by the partial melting and recrystallization of the internal broad crystalline phase. Specifically, when the programmed polymer is heated to 37 °C, a temperature positioned below the terminal melting point (43 °C), only the relatively smaller crystallites with lower melting temperatures melt. This partial melting allows the involved polymer chains to relax. Consequently, the polymer film macroscopically retracts toward its original shape, driven by the entropic elasticity of the robust crosslinked network. Upon cooling the polymer back to 0 °C, the remaining unmelted crystalline domains serve as robust nucleation sites, effectively guiding the recrystallizing polymer chains to pack predominantly along the initial stretching direction. Macroscopically, this directed crystallization manifests as the polymer elongating toward its temporary programmed shape. Therefore, dictated by this partial melting and directed recrystallization mechanism, the sample exhibits a robust and reversible bidirectional SME under cyclic thermal stimuli.

To visually demonstrate this remarkable reversible SME, the autonomous folding and unfolding behaviors of the samples were recorded during alternating thermal cycles between 0 °C and 37 °C. As presented in Figure 6c, the PEG-BA-20 film was first programmed by being completely folded at 70 °C and subsequently cooled to 0 °C, where this temporary folded shape was successfully fixed without any external constraint. Under stress-free conditions, when the ambient temperature was elevated to 37 °C, the film autonomously unfolded, tending to recover its original straightened state. Subsequently, upon cooling back to 0 °C, the film spontaneously actuated, bending back toward its temporary folded state. The reversible bending angle achieved during this 0 °C to 37 °C thermal cycle was calculated to be approximately 97°. Remarkably, this spontaneous angular switching capability of the film could be repeated continuously over multiple heating and cooling cycles without exhibiting any actuation attenuation.

### 3.5. Application Demonstrations of Bidirectional SMP in Thermoregulatory Textile

**Figure 7:**
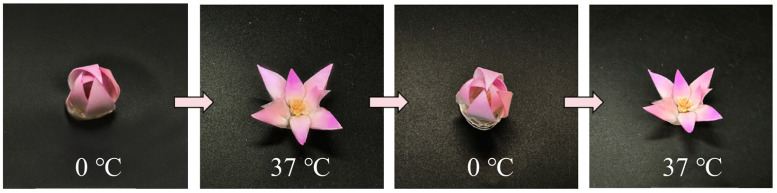
Stimulus-responsive petal simulated by bidirectional shape memory PEG-BA films.

As previously discussed, the petals of water lilies exhibit an autonomous diurnal actuation, naturally closing at night and dynamically opening at daytime. We hypothesized that the bidirectional shape memory PEG-BA film could be harnessed to simulate this autonomous flowering behavior. To validate this concept, a heterogeneous bilayer actuator, comprising an upper layer of physically inert PET tape and a lower layer of the programmed PEG-BA film, was precisely cut into petal-like architectures and assembled into a biomimetic flower. The thermo-responsive bending behavior of this actuator was systematically evaluated across a temperature range (0 to 37 °C) within a controlled environmental chamber (Figure 7). Upon exposure to a 0 °C environment, the artificial petals progressively bent inward, resulting in a completely closed floral structure in 15 s. This behavior occurred because the underlying programmed PEG-BA film underwent SME-driven elongation in cold conditions, while the upper PET tape maintained its dimensional stability, effectively forcing the petal to curl inward. Conversely, as the temperature gradually increased to 37 °C, the reverse SME triggered the macroscopic contraction of the PEG-BA film, compelling the petals to unfold outward with recovery time of 10 s. Evidently, under alternating cold–hot thermal cycles, the biomimetic flower successfully achieved a fully reversible “bloom-to-closure” actuation, mimicking the dynamic physiological characteristics of natural water lilies.

**Figure 8:**
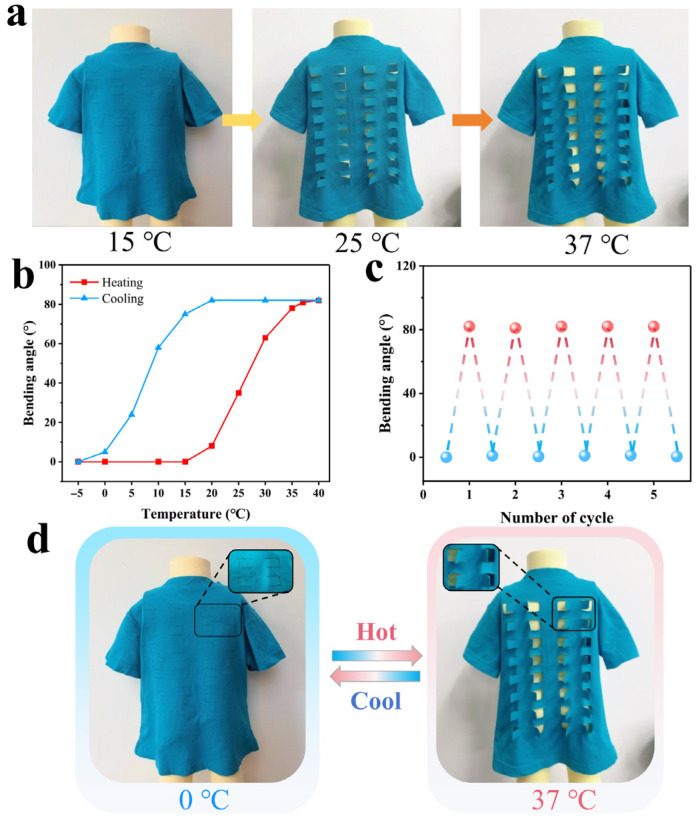
Temperature responsiveness of thermoregulatory textile. (**a**) Environmentally adaptive flaps in thermoregulatory textile under different temperatures, (**b**) bending angles of the flaps in response to environmental temperature changes, (**c**) cyclic bending angles and (**d**) corresponding photos of the flaps in thermoregulatory textile.

The reversible dynamic actuation of bidirectional SMPs holds profound potential for the development of temperature-adaptive garments. This technology enables the efficient regulation of human body heat dissipation, thereby effectively mitigating the thermal discomfort caused by sudden ambient temperature fluctuations. As a proof-of-concept, a prototype of thermoregulatory textile was engineered utilizing a conventional textile integrated with dynamic, temperature-responsive adaptive vents. The actuation mechanism was realized by securely attaching the temperature-responsive PEG-BA film onto the rectangular flaps of the fabric. The dynamic responsiveness of these smart textiles was quantitatively monitored under gradient ambient temperatures. Figure 8a displays the optical images of the smart clothing prototype subjected to dynamic thermal environments. It was visually confirmed that when the initially flat clothing was exposed to a gradually warming environment, the adaptive vents began to autonomously open at approximately 20 °C, eventually achieving a maximum bending angle of ~82° at 37 °C (Figure 8b). In this actuated state, excess heat generated by the human body is efficiently dissipated through the opened vents, thereby delivering a pronounced cooling effect to the wearer. Conversely, during the cooling process, the reversible vents initiated their spontaneous closure at around 15 °C and achieved a nearly fully closed configuration at 0 °C. This structural recovery significantly attenuates convective heat loss, providing a warming effect for the body. Furthermore, this macroscopic actuating behavior exhibited exceptional cyclic stability, with the bending–recovering performance remaining constant over multiple thermal cycles (Figure 8c and Video S1). In a word, the reversible opening and closing of these engineered vents effectively modulate human heat dissipation and retention across varying microclimates. For instance, when a wearer transitions from a cool, air-conditioned indoor setting to a hot outdoor environment, or vice versa, the autonomous structural adaptation of the garment significantly elevates personal thermal comfort.

## 4. Conclusions

In summary, we have developed a bidirectional SMP-driven thermoregulatory textile capable of autonomously modulating body-heat dissipation in response to fluctuating ambient temperatures. The core bidirectional SMP network was successfully constructed by crosslinking PEG precursors with a broad molecular weight distribution, and its properties were further tuned by incorporating BA components for desirable macroscopic softness. Consequently, the resulting PEG-BA network not only exhibited a broad crystal size distribution essential for continuous and progressive actuation, but also possessed a highly tunable elastic modulus owing to the introduction of highly flexible PBA chains. This endows the polymer with exceptional softness, perfectly matching that of conventional wearable textiles. Upon exposure to cyclic heating and cooling stimuli between 0 °C and 37 °C, the PEG-BA polymer exhibited a remarkable bidirectional SME, achieving a reversible actuation strain of 15.5%. Furthermore, as a proof-of-concept, we demonstrated thermoregulatory clothing with adaptive vents driven by the engineered PEG-BA polymer. This smart apparel could autonomously open and close these vents to effectively manage human heat dissipation. Notably, the dynamic temperature-responsiveness of the prototype exhibited no actuation attenuation over multiple heating–cooling cycles, demonstrating its exceptional dynamic stability. The proposed bidirectional SMP-based thermoregulatory textile significantly improves wearer comfort under fluctuating environmental conditions, showing tremendous potential for next-generation, passive thermal management wearable applications.

## Figures and Tables

**Table 1:** Thermal properties of the crystalline structure for the PEG-BA networks.

Sample	Run	T_onset_ (°C)	T_p_ (°C)	T_end_ (°C)	∆H (J/g)	X (%)
Pure PEG	Heating	13.6	41.5	46.8	57.8	28.2
	Cooling	25.7	7.0	−0.4	58.2	28.4
PEG-BA-10	Heating	10.6	39.2	45.0	58.8	28.7
	Cooling	24.7	3.2	−5.5	59.0	28.8
PEG-BA-20	Heating	7.1	34.8	39.7	48.6	23.7
	Cooling	21.4	−1.2	−9.2	48.7	23.8
PEG-BA-40	Heating	−0.1	33.9	39.2	34.8	17.0
	Cooling	20.8	−7.9	−18.3	34.2	16.7

## Data Availability

The data reported in this work are available from the corresponding author upon reasonable request.

## Supplementary Materials

The following supporting information can be downloaded at: https://www.mdpi.com/article/10.3390/biomimetics11050345/s1, Video S1: eDmonstration of temperature response of thermoregulatory textile.
